# Epidemiology of Suicide and the Psychiatric Perspective

**DOI:** 10.3390/ijerph15071425

**Published:** 2018-07-06

**Authors:** Silke Bachmann

**Affiliations:** 1 Clienia Littenheid AG, Hauptstrasse 130, 9573 Littenheid, Switzerland; silke.bachmann@clienia.ch; Tel.: +41-71-929-6300; 2 Department of Psychiatry, Psychotherapy, and Psychosomatics, Faculty of Medicine, University Halle (Saale), Julius-Kühn-Strasse 7, 6112 Halle (Saale), Germany

**Keywords:** suicidality, suicide, worldwide, epidemiology, age, gender, mental disease, psychiatric illness

## Abstract

Suicide is a worldwide phenomenon. This review is based on a literature search of the World Health Organization (WHO) databases and PubMed. According to the WHO, in 2015, about 800,000 suicides were documented worldwide, and globally 78% of all completed suicides occur in low- and middle-income countries. Overall, suicides account for 1.4% of premature deaths worldwide. Differences arise between regions and countries with respect to the age, gender, and socioeconomic status of the individual and the respective country, method of suicide, and access to health care. During the second and third decades of life, suicide is the second leading cause of death. Completed suicides are three times more common in males than females; for suicide attempts, an inverse ratio can be found. Suicide attempts are up to 30 times more common compared to suicides; they are however important predictors of repeated attempts as well as completed suicides. Overall, suicide rates vary among the sexes and across lifetimes, whereas methods differ according to countries. The most commonly used methods are hanging, self-poisoning with pesticides, and use of firearms. The majority of suicides worldwide are related to psychiatric diseases. Among those, depression, substance use, and psychosis constitute the most relevant risk factors, but also anxiety, personality-, eating- and trauma-related disorders as well as organic mental disorders significantly add to unnatural causes of death compared to the general population. Overall, the matter at hand is relatively complex and a significant amount of underreporting is likely to be present. Nevertheless, suicides can, at least partially, be prevented by restricting access to means of suicide, by training primary care physicians and health workers to identify people at risk as well as to assess and manage respective crises, provide adequate follow-up care and address the way this is reported by the media. Suicidality represents a major societal and health care problem; it thus should be given a high priority in many realms.

## 1. Introduction

Every year, about 800,000 human beings die from suicide; in other words, every 40 s someone deliberate kills her/himself. The global annual mortality rate has been estimated by the World Health Organization (WHO) to be 10.7 per 100,000 individuals, with variations across age groups and countries [1].

Terminology concerning suicides and suicide attempts has often included hints at intentions, motivations, and outcome (e.g., attention-seeking, relief, self-punishment [2,3,4]). It is therefore recommended to stick to neutral notions such as suicide, completed suicide, suicide attempts, and suicidality. Exclusively being descriptive, the Diagnostic and Statistical Manual of Mental Disorders (DSM-5) [5] uses the terminology “non-suicidal self-injury” (NSSI) in addition to “suicide behavior disorder”, and “suicide”.

## 2. Methods

A very broad literature search was performed using the Internet. This provided proof of the fact that the most encompassing and qualitatively best data are presented by the WHO. Their databases cover 194 member states [6]; data is collected according to the same principles and is therefore comparable. WHO literature was hand-searched for more detailed information; the biomedical data bank PubMed was scanned for literature related to suicide in the context of medical problems entering suicid* AND attempt.

For various reasons, extended and assisted suicide are not part of this paper.

## 3. Epidemiology

### 3.1. Suicide Worldwide

Suicide is a worldwide phenomenon. As such, it has continued to be addressed by the World Health Organization (WHO) since 1950, i.e., only two years after its foundation [7]. Obviously, the WHO and its coworkers have presented the most comprehensive and unbiased data from its member states, which serve as a basis for this paper.

Globally, suicides are the second leading cause of premature mortality in individuals aged 15 to 29 years (preceded by traffic accidents), and number three in the age-group 15–44 years [8]. Upsettingly, in 2015, the vast majority—namely 78% of suicides—were completed in low- and middle-income countries (LMIC) (WHO 2015). 

**Figure 1:**
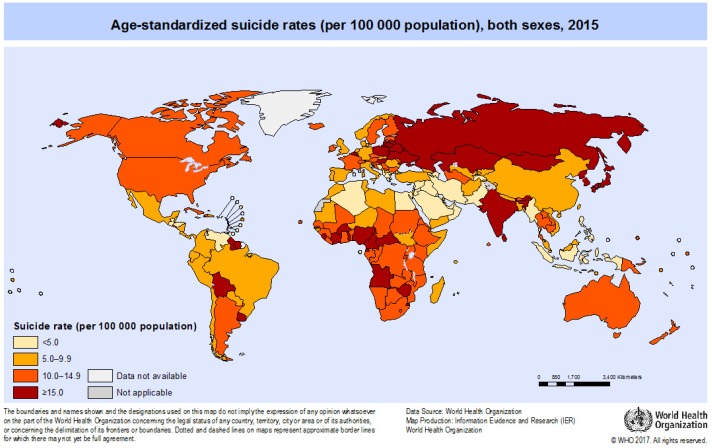
Suicide rates for both sexes around the world in 2015. Rates are standardized for age, because age profiles differ to a marked extent between countries. Reprinted with permission of WHO [10].

The global suicide mortality rate as depicted in Figure 1 amounts to 1.4%, ranging from 0.5% in African regions to 1.9% in the South-East Asia region [9]—please be aware of the fact that the WHO defines regions which do not completely overlap with geographic regions, e.g., the African region excludes the Eastern Mediterranean region/the Arabic countries.

To elucidate the uneven distribution of suicides between countries even further, some examples are presented. All rates refer to 100,000 inhabitants. The lowest rates, i.e., between 0 and 4.9, were found, for example, and in order of increasing rates, in Antigua and Barbuda, Barbados, Pakistan, Guatemala, Egypt, Syrian Arab Republic, United Arab Emirates, Indonesia, Iraq, Venezuela, Algeria, Jordan, Saudi Arabia, Philippines, Iran, Kuwait, Greece, and Morocco.

Suicide rates between 5.0 and 9.9 were documented in Mexico, Somalia, Bangladesh, Panama, Afghanistan, Libya, Tunisia, Peru, Nepal, Bosnia and Herzegovina, Brazil, Zambia, Kenya, Ghana, United Republic of Tanzania, Uganda, Kyrgyzstan, Viet Nam, Ecuador, Namibia, Italy, Macedonia, Ethiopia, Mozambique, Spain, United Kingdom, Turkey, Congo, Nigeria, Chile, and Singapore.

Rates between 10.0 and 14.9 existed in China, South Africa, Gabon, Norway, Ireland, Romania, Bhutan, Australia, Cambodia, Cameroon, Netherlands, Denmark, Lao People’s Democratic Republic, Canada, Slovakia, New Zealand, Iceland, Germany, Portugal, Czech Republic, Argentina, and USA.

The highest rates of ≥15 were found in Switzerland, Sierra Leone, Sweden, India, Democratic People’s Republic of Korea (North), Bulgaria, Thailand, Finland, Austria, France, Serbia, Bolivia, Estonia, Japan, Russian Federation, Belgium, Slovenia, Hungary, Latvia, Poland, Kazakhstan, Mongolia, Republic of Korea (South), Lithuania, and Sri Lanka.

When studying crude and age-standardized suicide rates according to WHO regions, both rates amount to 10.7 worldwide but fall apart in certain regions. The Eastern Mediterranean region has suicide rates of 3.8 and 4.3, the African region 8.8 and 12.8, the Americas 9.6 and 9.1, the Western Pacific region 10.8 and 9.1. South East Asia 12.9 and 13.3, and Europe 14.1 and 11.9 (all crude and age-standardized). 

Obviously, the European area presents the highest absolute or crude suicide rate, namely above the global suicide rate of 10.7 per 100,000 for both sexes. This is the case despite the fact that, since 1980, a drop in suicide rates was reached through preventive measures [11] and assisted suicides were taken out of the statistics. On the other hand, however, data quality is much better in comparison to other regions of the world, and fewer incidents are lacking in the statistics.

With respect to WHO regions or to continents, there have been some shifts regarding the highest suicide rates. When WHO initiated documentation, the highest rates were detected in Japan. The peak shifted to Eastern Europe (from the 1960s to 1980s: Hungary, from the 1990s to the 2010s: Lithuania), and to Asia thereafter [9] with China and India accounting for 30% of the absolute suicide numbers worldwide [12]. Vijayakumar et al. [13] state that even 54% of all suicides across the globe take place in the two mentioned countries. Another hypothesis holds that South Korea may become the front-runner soon [9]. Worldwide, WHO collaborators suppose that completed suicides will have risen to 1.53 million per year by the 2020 [8].

### 3.2. Age

In adolescents and young adults between 15 and 29 years of age, death from suicide reaches the highest absolute numbers. The US death statistics do not include death from suicide up to the age of 10 years. However, in the age group of 10 to 14 years, suicide is the third most common cause of death, representing the second most common cause up to the age of 34 years thereafter [14]. Overall, many more young than old individuals die from suicide, but the relative numbers per age group are up to eight times higher in the elderly [8]. Similarly, according to the WHO, children and adolescents up to the age of 15 years exhibit the lowest global suicide rates (per 100,000 inhabitants), which steadily increase thereafter until the age of 70 years or above (WHO 2014). Thus, an overall pattern is present; exemptions are discussed below [9].

The indicated overall pattern can clearly be detected in high-income European countries and South Korea [15]. In other countries, it is less obvious, and there are some exceptions. On the one hand, excessively high rates of suicide exist in young adults in low-income countries [16]. Inequality and a low quality of and access to health care most likely play a role. On the other hand, the highest rates of suicides arise in the middle-aged or old-aged groups [17,18] in other countries. In the US, in 2015, the highest suicide rates were present in the age-groups 45–64 years and 85 years and above (19.6 and 19.4) [19]. A similar distribution was reported from Australia, Canada, Greece, Ireland, Latvia, Lithuania, Netherlands, Norway, Poland, and the UK [15]. Thirdly, data from several African were published, which hint at the lowest age-related suicide rates in young people up to 25 years [20]. Yet, it has to be kept in mind that only 10% of these countries reported mortality data at all.

### 3.3. Gender

**Figure 2:**
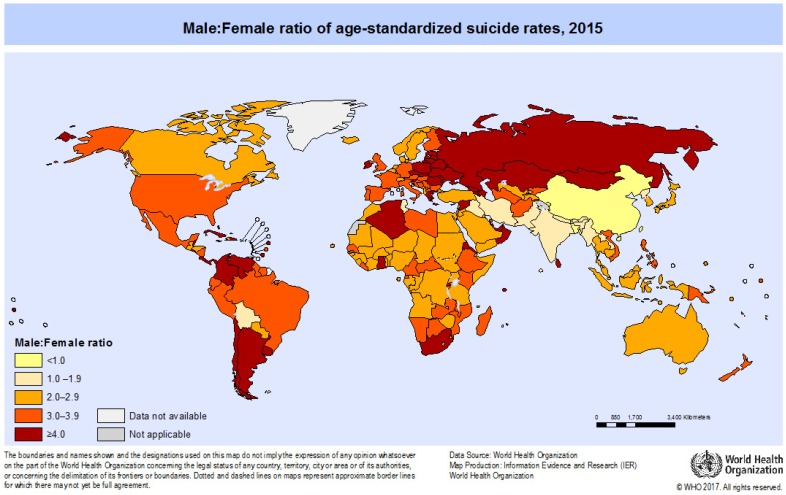
Global male:female suicide ratios per 100,000 in 2015 [23]. Reprinted from Global Health Observatory (GHO) data with permission of WHO.

The overall suicide rate of 10.7 per 100,000 population encompasses a male:female ratio of 1.7. Thus, men complete suicide almost twice as often as women [10]. However, comparing the information from 183 countries which was allocated in 2015, the male:female ratio varied from 0.8 in Bangladesh and China to 12.2 in St. Vincent and the Grenadines (see Figure 2).

A ratio below one means that the suicide rate of females exceeds that of males; this only exists in China and Bangladesh. Highest rates of suicides in women [10] were found in China (11.5), Angola (11.6), Japan and Belgium (12.4), and—among others—Sri Lanka (13.7), India (14.3), and both Koreas (North: 15.4; South: 16.4) [10]. Also, in South East Asia, unexpectedly high suicide rates in young females aged 15–29 were reported as the primary cause of death [21], whereas females aged 45 or above tend to die from suicide in the Western Pacific region.

The reverse, namely high suicide rates in men, was reported from Belarus (41.0), Guyana (42.4), Kazakhstan (46.8), Mongolia (47.8), Lithuania (58.0), and Sri Lanka (58.7) [22]. In terms of male:female ratio, the highest ratios were present in Belize (7.4), Saint Lucia (7.5), Seychelles (8.3), Jamaica (8.7), Panama (9.2), and St. Vincent and the Grenadines (12.2). Thus, men complete suicide up to 12 times more frequently than females. In the youngest age group, namely men aged 15–29 years, the highest suicide rate was present in South-East Asia, whereas in the European and Western Pacific regions, male suicide peaks arose in the groups 45 to 59 years, and 60 years and older, respectively [9]. Globally and since 1990, male suicides have accelerated considerably in number, which is not the case in females [6].

Altogether, absolute suicide numbers are not comparable between men and women, but age distributions follow the respective country’s pattern. Contrasting low and middle income countries (LMIC) with high-income countries (HIC) yields higher suicide rates for both young women and young men in the LMI compared to the HIC [7].

### 3.4. Socioeconomics

Socioeconomic variables strongly influence rates of suicide mediated by their being co-determinants of risk and, of course, of mental disorders. Several groups of determinants have been defined [24]:

(a) Demographic parameters: age, gender, ethnicity, and related parameters;
(b) Social status: low income, income inequality, unemployment, low education, and low social support;
(c) Social change: in a societal realm, e.g., urbanization, or on an individual basis, e.g., change in income;
(d) Neighborhood: inadequate housing, overcrowding, violence and others;
(e) Environmental incidences: climate change, natural catastrophe, war, conflict, and migration.

Many reasons for the high numbers of suicide in LMIC are included here. Seventy-eight percent of all suicides worldwide occur in LMIC [7,25], and the global rate has risen by 60% in the past 45 years, while during the same period of time Western countries saw a significant decrease [1].

Income, for example, is a factor which may be subject to change, more so than environmental incidences, and this is opposed to demographic parameters. Low income or unemployment and poverty constitute conditions which may lead to self-harm or suicide; vice versa, suicides may lead to loss of productivity and income, and thus to an increase in a family’s poverty. This vicious circle has been reported from countries or regions with varying gross domestic products including the US, Canada, Japan, 29 European countries, Iran, Chile, Sri Lanka, South and South-East Asia [26,27,28,29,30,31,32,33,34]. The crucial factor may well be inequality. Most likely, the influence of unfavorable socioeconomic factors play a more pivotal role in the lower income groups of each single country no matter whether they are high, middles, and low income countries absolutely speaking. Along these lines, the availability of health care not only differs between countries, but wherever present, education, and income determine its utilization [35].

High suicide rates also represent a financial burden to a society—mostly so, when young and middle-aged men, who are about to start or have just started their professional and family lives, complete suicide [7]. In the US, in the early 2010s, the costs per each single suicide were estimated to be over $1 million [36], whereas approximations from Ireland, Scotland, and New Zealand lay between $2.1 and $2.5 million [37,38,39]. In the literature on income, income equality and their relationship to suicides, only Bantjes et al. [26] included information from low-income countries such as sub-Saharan Africa, which made up 6% of the numbers. This opens up a huge gap between the overall numbers of suicides in the LMIC (78% of suicides worldwide) and the knowledge on costs within the respective societies [26].

### 3.5. Special Groups

Certain groups deserve special mention because they are at an even higher risk for suicide: the police, firefighters, and other first-line responders [40] as well as individuals in the army [41,42], incarcerated persons or those in high security hospitals (rates in men 7 times and in women 40 times higher than overall rates) [43], minorities [44], homeless people [45], refugees and asylum seekers [46,47]. Diverging results, however, arise from the literature on migrants: the suicide risk is said to increase [48,49], to remain stable [50], or to decrease [51] with migration, which may be related, among others, to the socioeconomic situation in the country of settlement [52]. A number of authors claim that ethnicity, country of origin as well as country of settlement influence suicide risk [53,54,55,56], not least because cultural differences between the countries may cause intergenerational as well as intrapsychic conflicts [55].

Also, lesbian, gay, transgender, and bisexual individuals are said to be at elevated suicide risk [57]; suicide attempts among transgender individuals reach 30–50% in some countries [58].

Attitudes, values, and beliefs strongly influence a possible decision to end one’s life [59]. As Islam forbids suicide, reported numbers from Muslim contexts are low. Where Hinduism or (secularized) Christianity are present, suicide rates circle around 10:100,000 (e.g., Italy 11.2, India 9.6). Clearly, higher suicide rates prevail in Buddhist Japan and atheist China, namely up to 17.9% and 25.6%, respectively [8]. Overall, care should be taken when looking at these numbers (as many others) as there may be mis- or underreporting. Culture in a broader sense and its impact on suicidal behavior, suicides, and prevention was highlighted by several authors [60,61,62].

Moreover, suicide rates are related to geography (e.g., amount of daylight) [63,64,65], to the presence of examples in the social context, i.e., “Werther effect” or copy-cat suicide [66,67,68], manmade factors such as legislation on suicide (e.g., suicidal behavior is illegal in several countries), domestic violence, and forced marriage [69].

### 3.6. Methods of Suicide

The latest overview of suicide methods goes back to 2008 [70] where authors distinguished hanging, drowning, falls, pesticide poisoning, other poisoning, firearms, and others. Differences regarding methods of suicide appear between regions more so than between countries. Regarding Africa: South Africans mostly hang themselves (69% in males; 41% in females); poisoning with pesticides and medication was the second common method used (35%, mostly females). In a review [20] of sub-Saharan Africa, data from 16 of 53 African countries only and thus 60% of the population had been obtained. This data mostly covered the larger cities. Suicide methods were available from 10 countries. The prevalence was highest for hanging and poisoning and clearly varied between countries (hanging 8–70%; poisoning 8–83%). A third method was the use of firearms (0–32%). No information is available with respect to possible gender effects.

The Americas: Suicides in the US most commonly occur by firearms, by males in 61% and by females in 36%; women also die from poisoning at a rate of 31%. Firearms were not used as often in the other American countries where both sexes rather completed suicide by pesticide poisoning (males: 0.4% in Canada to 86% in El Salvador; females: 1% in Canada to 95% in El Salvador) and hanging (males: 8% in El Salvador to 77% in Chile; females: 5% in Nicaragua to 63% in Chile). Obviously, outside the US, there are large differences between countries [70].

Asia: In the Asian region, people mostly chose hanging (23% in Hong Kong, 69% in Japan, 92% in Kuwait), with the exception that men from Hong Kong more often ended their lives through falls (43%) and other non-specified methods (23%). Both methods also prevailed in women from Hong Kong (48%, respectively 23%). In the other Asian countries, females lost their lives by hanging (26% in South Korea to 60% in Japan) or by pesticide poisoning (4% in Japan to 43% in South Korea). In Asia, on the whole, hanging and poisoning prevailed as methods of suicide [21], but Korea and the South-East Asian Region constitute exceptions with a higher prevalence of hanging and falls [71].

Europe: European males most commonly died by hanging (33% in Finland to 91% in Poland) with the exception of Swiss males who take their firearms (34%) home between the phases of mandatory military service. Firearm use in male suicide is the second common method in Finland, Norway, France, Austria, and Croatia (21–27%), whereas falls commonly were chosen in Luxembourg, Spain, and Malta (18–22%). Other, non-specified methods prevailed in Iceland, Denmark, UK, the Netherlands, and Georgia (20–33%). In European females, hanging (15% in Luxemburg to 83% in Lithuania) and other poisoning (7–43%) were methods of choice, but also falls (Luxemburg: 29%, Spain: 37%, Malta: 57%). Women in Moldova and Portugal poisoned themselves with pesticides (18% and 24%); in Georgia, other non-specified methods were reported as the main method (34%).

Australia and New Zealand: hanging predominated in males (45%, 48%), with other methods representing the second common method (both 29%), followed by firearms (12%, 11%). Females also chose hanging (36%, 43%) and other, non-specified methods (25%, 24%); the third position was taken by poisoning (27%, 20%).

Data from the Western Pacific Region were not available.

Recently, an article on methods of suicides in children and adolescents appeared. Authors reviewed 101 countries [72]. Overall, due to acceptability and availability, methods do not differ from those of adults in the same area. Hanging most frequently underlay unnatural deaths across age, gender, and region. The second most common methods were poisoning by pesticides in females and firearms in males.

Completing this section, the following list gives methods of suicide in the order of decreasing fatality: firearms (83%), drowning (66%), hanging (61%), gas poisoning (42%), falls/jumps (35%), poison ingestion (1.5%), cutting (1.2%), and other methods (8%) [73].

### 3.7. Suicide Attempts

Suicide attempts are included in the broader definition of self-harm, which means self-inflicted physical harm with or without an intention to die, the latter being taken into account.

Across the world, there is little data on suicide attempts and, if present, the quality is low due to a lack of reliable statistics, which again relates to under-, mis-, or non-diagnosing and reporting. The WHO does not receive information from any country in the world on this topic (Lee et al., 2017), although at least data from emergency rooms/somatic hospitals could be obtained as well as some self-reports [24].

According to the experts in the field, the number of suicide attempts is 10–30 times higher compared to completed suicides [19,74,75,76]. Numbers in the USA amount to as high as to 100–200 attempts per suicide in adolescents 15–24 years old [77]. There is little doubt that completed suicides are encountered more often in males than in females, with the exception of China and Bangladesh (see “gender”), where women attempt suicide 2–3 times more often than men. As for completed suicides, being young, widowed or divorced, and suffering from a psychiatric disorder depict overall risk factors for suicide attempts [78,79]. These criteria do not differ from completed suicides as is the case with methods used. However, increasing numbers of suicide attempts raise the risk of dying [24] and they are the most relevant risk factor for a completed suicide [7].

## 4. Suicide and Mental Illness

Psychiatric diseases account for a large majority of suicides and suicide attempts; numbers are at least 10 times as high as in the general population. The reported percentage of completed suicides in this context ranges between 60% and 98% of all suicides [8,80,81,82,83]. Many of the remaining incidents are related to problems concerning finances, relationships, and corresponding crises. However, other causes are discrimination [58], violence [84,85,86], terror, and war [87,88,89].

In the beginning of the 21st century, the highest mortality of unnatural causes globally was due to depression (30%), followed by substance-use related disorders (18%), schizophrenia (14%), and personality disorders (13%) [80]. However, in- and outpatients differed. Whereas 45% of inpatient suicides were preceded by schizophrenia and organic mental disorders, 32% of outpatients’ suicides occurred in the context of depression, and substance-related, somatoform, anxiety, and adjustment disorders. Depression was present in both groups, but rates differed [90].

An overall suicide rate of 147 per 100,000 inpatient years was given in a meta-analysis (Walsh et al., 2015). Being related to the general population’s suicide rate and to shorter stays in the hospital, suicide rates of inpatients lately increased. Single studies gave numbers in the range of 76 per 100,000 admissions in Germany [91] to 116/167 (women/men) per 100,000 in a study from Japan [92], and to 368 per 100,000 in an Australian study [93]. Recently, it has been speculated that the inpatient-status by itself may be a risk factor [94], as the lifetime suicide risk is much higher than the 8.6% reported for never-hospitalized outpatients [81].

Special attention should be paid during the 4–12 weeks following discharge from inpatient treatment, when suicide rates rise [95,96,97,98]. A further increase was stated for males and for individuals with a history of suicide attempts [97]. However, another study identified a diagnosis of schizophrenia, a longer stay in hospital, and previous suicide attempts as risk factors, but not suicidality prior to the index-admission [91]. Less is known with regard to outpatients because diagnoses often are unknown, unclear or omitted in the death certificate; unnatural causes of death may not be detected [92].

### 4.1. Suicide According to Selected Diagnoses

Patients suffering from organic mental disorders who previously underwent inpatient treatment complete suicide 10 times more often than outpatients [12]. Among ICD-10 F0 diagnoses, dementia constitutes a moderate risk if not more [99,100,101,102,103,104], including pre-emptive suicide [105,106].

Concerning substance use disorders (ICD-10 F1), there is some confusion in the literature, because they are sometimes separated from mental disorders, although they are listed in ICD-10 chapter F1 [82,107]. In outpatients, substance-related disorders represent the second most common reason (22.4%) for completing suicide, even in 12–18 year-olds. The incidence in inpatients is twice as high. In both groups, alcohol constitutes the frontrunner, with permissive cultures having higher suicide rates related to alcohol than restrictive ones [71,108,109]. The suicide risk in alcoholism rises by 2–3.4% when previous treatment had taken place. Further related risk factors are previous suicide attempts, male gender, and older age [110,111,112]. On the other hand, suicide risk is even higher in adolescence, probably mediated by alcohol-related neurological and psychological dysfunctions [113]. Additionally, alcohol may facilitate the consumption of other drugs, including illicit ones [114,115]. Alcohol intoxication needs special mention because it increases suicidality by itself, especially when related to adjustment disorders (e.g., bereavement) and depression [116]. Chronic use of and intoxication with other substances are comparable [111,117].

Speaking about absolute numbers of substance-influenced suicides, 40–85% of those occur after intake of alcohol and/or sedative-hypnotic drugs in otherwise non-consuming individuals [111,118]. Iatrogenic effects come into play in sedative-hypnotic drugs, but the rate of prescriptions is unclear as is the potential role of withdrawal symptoms e.g., [118,119].

Completed suicides rise by a factor of 14 in heroin consumers. Up to 35% of deaths related to heroin were said to be unnatural [120]. Cannabis did not constitute an independent risk factor, but cocaine and methamphetamine are related to suicide attempts in 20% of users and to suicidality in about two-thirds of users [111,121,122]. Among substance dependent individuals who seek treatment, 40% have a history of at least one suicide attempt [4].

Substance misuse and dependence frequently co-occur with other psychiatric illnesses [111], the disease onset of the latter may be preponed by substances [123,124].

In schizophrenia (ICD-10 F2), suicides are completed by at least 5–14% of all affected individuals [80,125,126] and attempts take place during the first years of illness in about 10% of patients [127]. In early onset schizophrenia, i.e., in adolescents, numbers of attempts and completed suicides exceed those of adult onsets [128]. Schizophrenia is the second most frequent diagnosis preceding inpatient suicide (20%), with a rate twice as high in comparison to outpatients [8]. Among others, the following factors add to the risk: depressive and hallucinatory symptoms [129,130], male sex, high premorbid IQ, feelings of guilt or anxiety, substance abuse, treatment delay, closeness to illness onset or to psychiatric inpatient treatment, number of psychiatric admissions, history of suicide attempts or non-suicidal self-harm [131,132,133,134,135].

Depression is the leading cause of death of suicide worldwide and is number two in years lived with disability (globally up to 11%) [136,137]. Half of all completed suicides are related to depressive and other mood disorders (ICD-10 F3); in comparison with healthy subjects, a 20-fold increased risk was reported [138,139]. Receiving treatment for depression is associated with the availability of treatment and the severity of depressive symptoms. Nevertheless, up to 50% of depressed individuals in high-income countries and up to 85% in low and middle-income countries (LMIC) seem to go without treatment for a period of 1 year [139]. Where individuals with severe depressive symptoms usually are admitted to an inpatient service, their suicide rates (21%) double those of outpatients [80]. Results of a Swedish national cohort resemble the global data; the study gave numbers in the range of 12–19% suicides in depressed inpatients [138].

Suicides linked to depression occur more in the elderly, a good proportion of whom experience psychotic symptoms [140,141]. As opposed to depression, suicide attempts and suicides in bipolar disorder concur with the first depressive episode, namely around the age of 25 years [142]. Illicit drug use may prepone disease onset including suicidality for up to 6 years; this effect has particularly been described in cannabis users, and suicidality thus may rise to 60% [124]. Other factors in bipolar patients’ completed suicide are male gender and a first-degree family history of suicide. Parameters associated with suicide attempts are female gender, younger age at illness onset, any substance or alcohol use, depressive polarity of the first or most recent episode, comorbidities such as anxiety disorder or cluster B personality disorders [143].

Mortality-related suicide in anxiety disorders (ICD-10 F4) amounts to 2.5% in inpatients and to 6% in outpatients [12]. While these numbers have remained stable during the past decades, suicides related to obsessive-compulsive disorders have moderately increased [144]. Posttraumatic stress disorders (PTSD) play a special role among diseases listed in this chapter. There is an increased incidence of PTSD worldwide through terrorism, wars, migration, and the like, and co-morbidities are common [81,145,146,147,148]. The suicidality rate is estimated to about 20% in this population with even higher numbers in adolescents [149,150,151,152].

Among the ICD-10 F5 diagnoses, the highest risk of suicide is related to eating disorders [153]. A study by the British National Health Service (NHS), which followed patients after discharge form hospital, found a standard mortality ratio of 7.8 for all eating disorders in the age group 15–24 years, amounting to 4.1 in bulimia nervosa and to 11.5 in anorexia nervosa. Respective numbers in an adult group aged 25–44 years were 10.7 for all eating disorders, 14.0 for anorexia nervosa, and 7.7 for bulimia nervosa [154].

Lastly, personality disorders (ICD-10 F6) deserve mention as a high-risk group, with 15% of inpatient and almost 12% of outpatient suicides [12,80]. In this varied group of conditions, there are, of course, subgroups. Borderline personality disorder deserves the highest mention, with suicide rates ranging from 3% to 9% [80,155,156,157]. In addition, chronic suicidality poses a major problem for therapists in borderline personality disorder patients [155,158,159]. The second highest suicide risk in this group is associated with the narcissistic personality disorder [160]; although they are related to many suicide attempts, they account for less than 5% of completed suicides [2,161,162,163]. The remaining personality disorders account for a small percentage each.

### 4.2. Comorbidities

Each single comorbidity raises the risk of suicide, which holds true for all mental disorders. Combinations with psychotic disorders depict the highest risk (50% increase in inpatients), and mood disorders are second, especially when co-occurring with substance abuse or personality disorders [12,164,165].

In summary, suicides in outpatients are related to the following diagnoses: mood disorders, substance abuse, personality disorders, anxiety, and adjustment disorders including PTSD. Inpatients’ diagnoses associated with suicide are mood disorders, schizophrenia, and organic mental disorders. Comorbidities increase suicide risk in both settings [166,167,168].

### 4.3. Suicide and Physical Illness

Not only is the prevalence of suicide attempts and suicides elevated in individuals with psychiatric illness, but also in the context of physical health problems. The literature frequently mentions potentially lethal disorders such as cancer and HIV-infection. The number of suicides doubles in individuals who are diagnosed with cancer, irrespective of comorbidities such as substance use and depression [169,170,171]. While the relationship between cancer and suicide was studied in high-income countries (HIC), the one between HIV-infection and suicide was reported from LMIC [172,173,174,175]. Ultimately, any chronic disease may be associated with an elevated risk of suicide; among others, the literature lists multiple sclerosis, epilepsy, systemic lupus erythematodes, asthma, and hemodialysis for kidney failure [153,176,177,178,179]. An important issue in chronic physical disease is disability, which leads to an increase in suicidality (Rahman et al., 2014); traumatic brain or spinal cord injury [180,181], and post-stroke conditions [182] may be named. With an increasing degree of bariatric surgery, a consequential rise in suicides has been reported [183,184]. In physical as well as in mental illness, the number of suicides increases with every comorbidity. The mere fact of being hospitalized seems to increase the risk of suicide, as a Taiwanese study indicates: the occurrence of suicides in a general hospital was eight-times that of the general population [185]. Along these lines, Pompili et al. [186], studied suicide attempts in an emergency department. They found insomnia, be it related to underlying disorders or not, may be a precursor—and if so, attempts were undertaken by more violent methods. Thus, health problems in general may represent a major reasons for suicide [187].

Chronic pain deserves special mention because it overlaps with depression to a considerable degree. As causality is unclear [188], chronic pain may be regarded as a physical or a somatoform disorder, the latter being a mental condition. The relevance becomes obvious when looking at the high prevalence data, which are in the range of 10 to 55% in HIC [189], and the fact that the suicide rate is 2–3 times that of control subjects; however, the number of suicide attempts is even higher [190,191]. These numbers remain even when comorbid mental illness is accounted for [192].

## 5. Prevention

Countries and communities may influence suicide rates by measures of primary and secondary prevention. In HICs such as Germany, Japan, and Korea a decrease has been reached by preventive measures and addressing vulnerable groups [193] between 1990 and 2010 [194,195]. Since 2000, national prevention strategies have been established in 28 countries; these include primary and secondary preventive strategies in Europe (13 programs), the Americas (8 programs), Western Pacific (5 programs), South-East Asia (2 programs), and Africa and Eastern Mediterranian (0 programs) [7].

### 5.1. Primary Prevention

Mostly, a survey is the basis of any prevention, as is the case for example in the “European Multicentre Study on Suicidal Behaviour and Suicide Prevention” (MONSUE) [196]. 

The simplest measure towards suicide reduction is the blocking of access to respective means: poison, potentially poisonous medication such as paracetamol, bridges, firearms, and railways. Impressive examples are the barriers at the Golden Gate Bridge in San Francisco, the Empire State Building in New York City, and the Eiffel Tower in Paris [197], and the reduction of access to firearms [198], which all lead to drops in suicide rates. Comparable effectiveness was shown by data from 21 OECD countries [11]. Unfortunately, not all measures of blocking access to means of suicide prove successful as has been reported recently [199].

Also, the media disposes of an important leverage to influence suicide rates. Their reporting may or may not encourage copycat suicide. Several authors have suggested the implementation of guidelines for journalists [68,200], and the WHO has published respective sources for journalists [201].

Another measure to reduce suicide rates is community awareness programs. These usually imbed helplines and public education at workplaces and schools to increase knowledge and reduce stigma. Programs draw on lay gatekeepers such as clergy, teachers, and first-line responders who receive special training. Along these lines, involving and training laypersons or non-specialized health professionals is an important means of suicide prevention, assessment, and management where health care resources are scarce. Online and telephone counselling by trained volunteers has been established and is accepted worldwide for providing effective support [202,203].

Similarly, professionals in primary care should be trained [204,205]. It has been shown in HIC that most individuals who later completed suicide had seen a physician of mental health professional during the 12 months prior to their deaths [206,207]. 

Moreover, the importance of crisis intervention for help-seeking individuals has been demonstrated in two East-European countries, where a reverse correlation between suicide rates and the number of physicians was present [208,209]. This changed with an increasing number of health care professionals and an increasing treatment rate of depression. On the other hand, care should be taken not to misuse compulsory admissions, as there might be false positives and persons drawn to suicide by adverse experiences [210].

A special aspect of primary prevention refers to the internet and especially social media, which provide a multitude of information. Help-seekers will find abundant information and addresses of lay and professional support, which is certainly sensible. The same amount of information, however, is available for those who plan suicide: pro-suicide websites, blogs or chatrooms, which give instructions or support suicide pacts [211,212,213]. Thus, positive and negative aspects of social media/the internet exist equivalently, risks of usage should be addressed in awareness campaigns and barriers to pro-suicide sites discussed. It has to be kept in mind, also, that the current internet use mirrors only those you own computers, namely the English-speaking population of HIC and of the upper classes of LMIC.

### 5.2. Secondary Prevention

With respect to secondary prevention, the health care system is of utmost importance. A large number of 22–88% of suicide attempters, according to the respective culture, seek help afterwards by presenting to a hospital or to primary care [75]. Every health worker or gatekeeper should be aware of the fact and be trained to react adequately. Although present and evaluated in Europe [196], hospital-based surveillance to detect suicide attempts is still needed in the majority of LMIC [214].

Self-help groups for bereaved relatives and survivors of suicide attempts deserve positive mention since their importance and recognition has grown since 2000 [215].

## 6. Discussion

### Quality of Data

The WHO states that regular miscoding is related to causes of deaths which are stigmatized [216]. Thus, research on suicide and its results is shaped by global or regional caveats much more so than any other area of medicine. In the global realm, suicides as well as suicide attempts may not be correctly classified but looked at as an accident. This may be the case in car accidents, poisoning, and many other injuries. The WHO suspects that underreporting ranges between 20% and 100% and is rooted in beliefs, stigma, politics, and legislation (e.g., prosecution of suicide attempts in certain countries) [12].

Along these lines, a lack of knowledge among medical professionals represents another caveat and leads to the misdiagnosing of death as being accidental: drowning and submersion, falls, accidental poisoning by and exposures to noxious substances, exposure to smoke or fire, and assault. Understandably, diagnosing sometimes is incorrect due to a lack of information, as in drug overdosing. External causes of death shall be classified according to ICD-10 codes R, V-Y [217] by all means, independently of any intention. Likewise, if the respective information is available, injury codes should not be used in the case of death due to intoxication.

**Figure 3:**
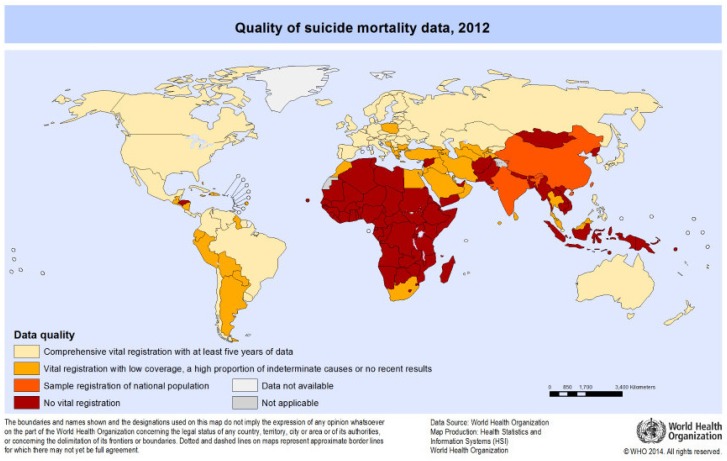
Quality of suicide mortality data, 2012 [218]. Reprinted with permission of WHO.

A review of 114 studies deduced that the higher the quality of single studies, the higher the number of the reported suicide rate [21]. Likewise, the assessment of suicide mortality by WHO in 2012 (then 172 member countries) found a good data quality of suicide reporting in 60 countries only, whereas an estimated 71% of suicides were completed in the remaining 112 countries. There was a clear link to financial resources: HIC dispose of better quality registers. This was the case in 39 HIC where 95% of presumably all suicides were captured as opposed to 8% of estimated suicides in 21 LMIC (see Figure 3).

WHO initiated another assessment in 2015 and specified five categories for the definition of data quality in its Mortality Database. The following was rated as high quality data: the presence of ICD codes or a cause list, average usability of data at least 80% since 2000, availability for at least 5 years prior to assessment [216]. Availability shall serve as an example of data quality: several, especially African countries, did not provide any data at all; the last available measurements stemmed from 1983 in the Falkland Islands, 1987 in Monaco, 1990 in Zimbabwe, 2003 in Bolivia, 2004 in Haiti, 2005 in Tajikistan, 2006 in Sri Lanka, 2007 in Azerbaijan, 2008 in Malaysia and Iraq, and 2009 in Montenegro (not conclusive) [15].

Obviously, it is very difficult to obtain reliable data on suicide rates. However, assuring rates of suicide attempts is nearly impossible, not least because a suicide attempt may not come to anyone’s attention, much less to the attention of the health care system. Nevertheless, the registration of suicides and suicide attempts is a desirable goal towards better prevention, detection, and intervention [8].

## 7. Conclusions

To summarize, the best available data on suicide and, less so, on suicide attempts are being presented and updated regularly by the WHO. Information on 194 countries leads to the assumption that suicide rates vary with the diversity of changing economic, social, cultural, and environmental factors as well as with age and gender. 

Also, globally, suicide rates are increased in individuals with chronic physical and mental illnesses, including abuse of alcohol and substances, and in those who have already attempted suicide. Across the world, the quality of data is low to medium due to under-, mis-, or non-diagnosing and reporting. Therefore, much less is known about suicide attempts; they probably outnumber suicides by 30 times.

Prevention is possible; therefore, the implementation of the respective measures is warranted worldwide. 

## Abbreviations and Explanations

HIC	high income countries
LMIC	low and middle income countries
Suicide rate	per 100,000 persons, where the weights are the proportions of persons in the corresponding age groups of the WHO standard population
Crude suicide rate	number of suicide deaths in a year (and a rate: geographic area), divided by the population (in this area) and multiplied by 100,000
Age-standardized suicide rate	a weighted average of the age-specific suicide rates
